# The Magnetohydrodynamic Stagnation Point Flow of a Nanofluid over a Stretching/Shrinking Sheet with Suction

**DOI:** 10.1371/journal.pone.0117733

**Published:** 2015-03-11

**Authors:** Syahira Mansur, Anuar Ishak, Ioan Pop

**Affiliations:** 1 Department of Mathematics and Statistics, Faculty of Science, Technology and Human Development, Universiti Tun Hussein Onn Malaysia, Parit Raja, Batu Pahat, Johor, Malaysia; 2 School of Mathematical Sciences, Faculty of Science and Technology, Universiti Kebangsaan Malaysia, UKM Bangi, Selangor, Malaysia; 3 Department of Mathematics, Babeș-Bolyai University, Cluj-Napoca, Romania; North China Electric Power University, CHINA

## Abstract

The magnetohydrodynamic (MHD) stagnation point flow of a nanofluid over a permeable stretching/shrinking sheet is studied. Numerical results are obtained using boundary value problem solver bvp4c in MATLAB for several values of parameters. The numerical results show that dual solutions exist for the shrinking case, while for the stretching case, the solution is unique. A stability analysis is performed to determine the stability of the dual solutions. For the stable solution, the skin friction is higher in the presence of magnetic field and increases when the suction effect is increased. It is also found that increasing the Brownian motion parameter and the thermophoresis parameter reduces the heat transfer rate at the surface.

## Introduction

Nanofluid refers to dispersion of nanoparticles in the base fluid. The inclusion of nanoparticles enhances thermal conductivity as reported by Masuda et al. [1]. Choi and Eastman [2] discovered that the addition of less than 1% of nanoparticles doubles the heat conductivity of the base fluid. We mention to this end that the paper by Wong and Leon [3] includes automotive, electronics, biomedical and heat transfer applications besides other applications such as nanofluid detergent. Saidur et al. [4] presented some applications of nanofluids in industrial, commercial, residential and transportation sectors based on available literatures. The capability of nanofluids to enhance thermal conductivity has attracted the interest of fluid dynamics community to conduct further studies.

Two models have been constantly used by researchers to study the behaviour of nanofluids, namely the Tiwari-Das model [5] and Buongiorno model [6]. While Tiwari-Das model highlights the volumetric fraction of nanoparticles which is used by Ul Haq et al. [7], Nadeem et al. [8] and Sheikholeslami et al. [9], Buongiorno model focuses on Brownian motion and thermophoresis effects. Many researchers then include the effect of Brownian motion and thermophoresis in their studies such as Nield and Kuznetsov [10], Kuznetsov and Nield [11–13], Khan and Pop [14], Bachok et al. [15, 16], Khan and Aziz [17], Ul Haq et al. [18], Haq et al. [19], Nadeem and Ul Haq [20, 21] and Nadeem et al. [22] among others. Sheikholeslami et al. [23, 24], Akbar et al. [25, 26] and Ellahi [27] study the flow of nanofluid with magnetic effects where different methods are used to solve the problems. Furthermore, Akbar et al. [28] also study the blood flow of a nanofluid by using homotopy perturbation method. Boundary layer flow of a nanofluid moving through several mediums such as cylinders, rectangular ducts and rotating plates are discussed by Zeeshan et al. [29] Ellahi et al. [30], Nadeem et al. [31] and Sheikholeslami et al. [32]. Notably, Ellahi et al. [33] produces series solutions of a non-Newtonian nanofluid where Reynold’s and Vogel’s model are used to represent variable viscosity.

The boundary layer flow over a stretching sheet is significant in applications such as extrusion, wire drawing, metal spinning, hot rolling etc. [34]. Wang [35, 36], Mandal and Mukhopadhyay [37], Gupta and Gupta [38], Andersson [39], Ishak et al. [40] and Makinde and Aziz [41] are among various names whose papers on stretching sheet are published. However, to complement the study of stretching sheet, Miklavčič and Wang [42] then begin the study of flow over a shrinking sheet in which they observed that the vorticity is not confined within a boundary layer and a steady flow cannot exist without exerting adequate suction at the boundary. However, according to Wang [43], a stagnation flow may also be considered so that the velocity of the shrinking sheet is confined in the boundary layer. As the studies of shrinking sheet garner considerable attention, these findings prove to be crucial to these researches. Numerous studies on these problems have been conducted by researchers such as Fang et al. [44], Bachok et al. [45], Bhattacharyya et al. [46], Lok et al. [47, 48], Zaimi et al. [49] and Roşca and Pop [50] among others.

Motivated by the above-mentioned researches, this paper aims at studying the magnetohydrodynamic (MHD) stagnation point flow of a nanofluid over a stretching/shrinking sheet with suction effect at the boundary which is the extension of a paper done by Ibrahim et al. [51]. MHD is the study of the dynamics of electrically conducting fluids such as plasmas, liquid metals and electrolytes The dependency of the skin friction coefficient and the local Nusselt number on five parameters, namely the stretching/shrinking, magnetic, velocity ratio, Brownian motion and thermophoresis parameters is the main focus of the present investigation. Numerical solutions are presented graphically and in tabular forms to show the effects of these parameters on the skin friction coefficient and the local Nusselt number. number.

### Mathematical Formulation

**Fig 1:**
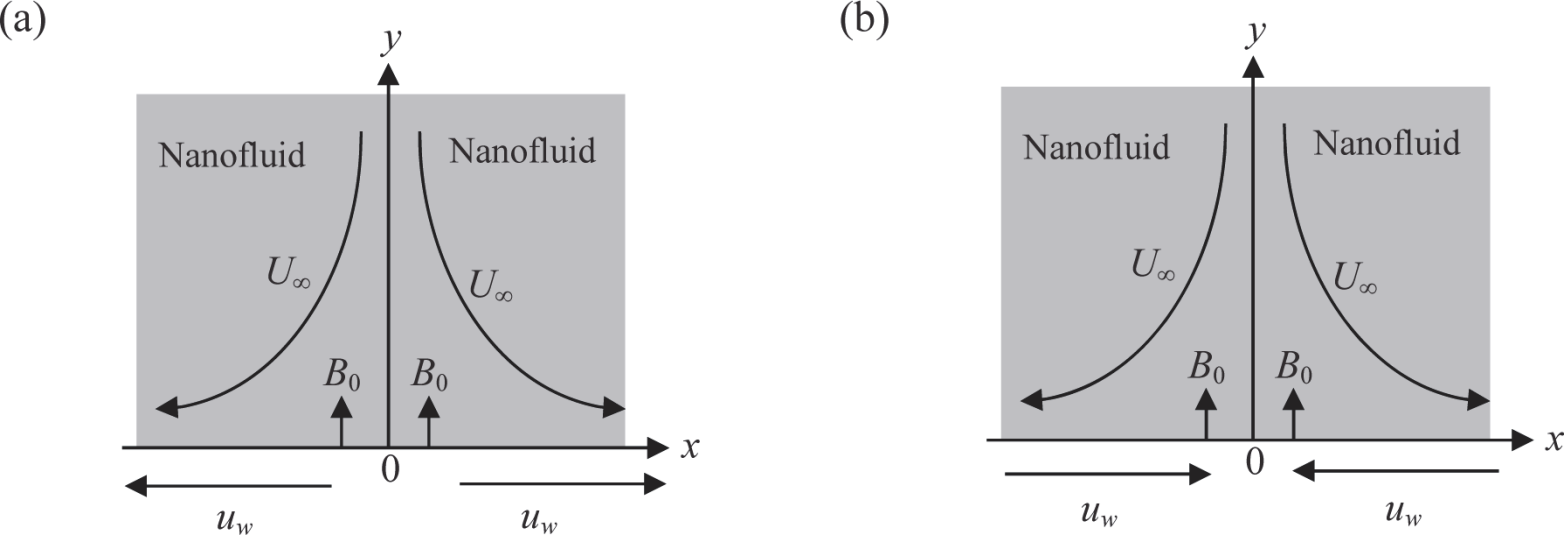
Geometry of the problem for (a) stretching sheet [49] and (b) shrinking sheet.

We examine the boundary layer flow of a nanofluid towards a stagnation point on a permeable stretching/shrinking surface, kept at a constant temperature *T*_*w*_ and concentration *C*_*w*_, at *y* = 0 as shown in Fig. 1, where the *x*− and *y*− axes are taken along and normal to the stretching/shrinking surface. It is assumed that the free stream velocity is *U*_*∞*_(*x*) = *bx* and the plate is stretched/shrunk with the velocity *u*_*w*_ *(x) = cx* where *b* and *c* are positive constants. It is also assumed that the constant mass flux velocity is *v*_0_ with *v*_0_ < 0 for suction and *v*_0_ > 0 for injection. The ambient values of the temperature and nanoparticle fraction are taken as *T*_*∞*_ and *C*_∞_, respectively. In view of thermal equilibrium, there is no slip between the base (or ordinary) fluid and suspended nanoparticles. Furthermore, the flow is subjected to a constant transverse magnetic field of strength *B* = *B*_0_ which is assumed to be applied to the positive *y*-direction. Under these assumptions and the boundary layer approximations, the unsteady governing continuity, momentum and energy boundary layer equations are (see Ibrahim [51])

$$\frac{\partial u}{\partial x}+\frac{\partial v}{\partial y}=0,$$ (1)

$$\frac{\partial u}{\partial t}+u\frac{\partial u}{\partial x}+v\frac{\partial u}{\partial y}=U_{\infty }\frac{d\;U_{\infty }}{d\;x}+\nu \frac{\partial^{2}u}{\partial y^{2}}+\frac{\sigma B_{0}^{2}}{\rho_{f}}\left(U_{\infty }-u\right),$$ (2)

$$\frac{\partial T}{\partial t}+u\frac{\partial T}{\partial x}+v\frac{\partial T}{\partial y}=\alpha \frac{\partial^{2}T}{\partial y^{2}}+\sigma \left[D_{B}\frac{\partial C}{\partial y}\frac{\partial T}{\partial y}+\frac{D_{T}}{T_{\infty }}{\left(\frac{\partial T}{\partial y}\right)}^{2}\right],$$ (3)

and

$$\frac{\partial C}{\partial t}+u\frac{\partial C}{\partial x}+v\frac{\partial C}{\partial y}=D_{B}\frac{\partial^{2}C}{\partial y^{2}}+\frac{D_{T}}{T_{\infty }}\frac{\partial^{2}T}{\partial y^{2}}$$ (4)

where *u* and *v* are the velocity components along the *x*- and *y*-axis respectively, *U*_*∞*_ is the free stream velocity, *t* is time, *T* is the fluid temperature, *α* is the thermal diffusivity, *ν* is the kinematic viscosity, *D*_*B*_ is the Brownian diffusion coefficient, *D*_*T*_ is the thermophoresis diffusion coefficient and *C* is the nanoparticle volume fraction. Furthermore, *σ* = (*ρc*)_*p*_/(*ρc*)_*f*_ is the ratio between the effective heat capacity of the fluid with *ρ*_*f*_ and *ρ*_*p*_ being the density of the fluid and the density of the particles respectively and *c*_*f*_ and *c*_*p*_ denote the specific heat of the fluid and the particle at constant pressure, respectively.

The equations are subjected to the boundary conditions

$$\begin{array}{l} t<0:\;u=v=0,\;T=T_{\infty },\;C=C_{\infty }\;\text{for any}\;x,y,\\ t\geq 0:\;v=v_{0},\;u=\lambda u_{w}(x),\;T=T_{w},C=C_{w}\;\text{at}\;y=0,\\ \;u\to U_{\infty }(x),\;T\to T_{\infty },\;C\to C_{\infty }\;\text{as}\;y\to \infty \end{array}$$ (5)

where *λ* is the stretching parameter with *λ* > 0 for stretching and *λ* < 0 for shrinking, and the subscript *w* denotes the values at the solid surface. We mention that we have followed in this paper the boundary conditions from the paper by Kuznetsov and Nield [11]. However, very recently Kuznetsov and Nield [13] have mentioned that the major limitation of the model in the paper [11] was active control of nanoparticle volume fraction at the boundary, while in the paper [13], the nanofluid particle fraction on the boundary is passively rather than actively controlled. It is a statement where, with thermophoresis taken into account, the normal flux of nanoparticles is zero at the boundary. This makes the model physically more realistic.

### Steady-state flow

In order to solve the steady-state flow (*∂*/*∂t* = 0) of Eqs. (1) to (4) with the boundary conditions (5), we introduce the following similarity variable

$$\begin{array}{l} \psi =\sqrt{c\nu }\;x\;f(\eta ),\;\theta (\eta )=(T-T_{\infty })/(T_{w}-T_{\infty }),\\ \;\phi (\eta )=(C-C_{w})/(C_{w}-C_{\infty }),\;\eta =\sqrt{\;c/\nu }\;y \end{array}$$ (6)

where *ψ* is the stream function, which is defined as *u* = *∂ψ* /*∂y* and *v* = −*∂ψ/∂x*. Thus, we have

$$u=c\;x\;f'(\eta ),\;v=-\sqrt{c\nu }\;f(\eta )$$ (7)

where prime denotes differentiation with respect to *η*.

Substituting variables (5) into Eqs. (2) and (3) for the steady-state flow (*∂/∂t = 0*), we obtain the following ordinary differential (similarity) equations

$$f^{\prime \prime \prime }+ff^{\prime \prime }-{f^{\prime }}^{2}+M\left(A-{f^{\prime }}^{2}\right)+A^{2}=0,$$ (8)

$$\frac{1}{\mathrm{Pr}}\theta^{\prime \prime }+f\theta^{\prime }+Nb\phi^{\prime }\theta^{\prime }+Nt{\theta^{\prime }}^{2}=0,$$ (9)

and

$$\phi^{\prime \prime }+Lef\phi^{\prime }+\frac{Nt}{Nb}\theta^{\prime \prime }=0$$ (10)

subject to the boundary conditions (4), which become

$$\begin{array}{l} f(0)=S,\;f'(0)=\lambda ,\;\theta (0)=1,\;\phi \left(0\right)=1,\\ f^{\prime }\left(\eta \right)\to A,\;\theta (\eta )\to 0,\;\phi (\eta )\to 0\;\text{as}\;\eta \to \infty . \end{array}$$ (11)

Here, Pr is the Prandtl number, *M* is the magnetic parameter, *S* is the constant mass transfer parameter with *S* > 0 for suction and *S* < 0 for injection, *A* is the velocity ratio, *Le* is the Lewis number, *Nb* is the Brownian motion parameter and *Nt* is the thermophoresis parameter, which are defined as

$$\begin{array}{l} \mathrm{Pr}=\frac{\nu }{\alpha },\;Nb=\frac{\sigma D_{B}(\phi_{w}-\phi_{\infty })}{\nu },\;Nt=\frac{\sigma D_{T}(T_{w}-T_{\infty })}{\nu T_{\infty }},\;Le=\frac{\nu }{D_{B}},\\ S=-\frac{v_{0}}{\sqrt{a\nu }},\;M=\frac{\sigma B_{0}^{2}}{\rho_{f}c},\;A=\frac{b}{c}. \end{array}$$ (12)

The quantities of physical interest are the skin friction or shear stress coefficient *C*_*f*_, and the local Nusselt number *Nu*_*x*_, which are defined as

$$C_{f}=\frac{\tau_{w}}{\rho u_{w}^{2}(x)},\;Nu_{x}=\frac{x\;q_{w}}{k(T_{f}-T_{\infty })}$$ (13)

where *τ*_*w*_ is the skin friction or shear stress along the plate and *q*_*w*_ is the heat flux from the plate, which are defined as

$$\tau_{w}=\mu {\left(\frac{\partial u}{\partial y}\right)}_{y=0},\;q_{w}=-k{\left(\frac{\partial T}{\partial y}\right)}_{y=0}.$$ (14)

Using (5), (10) and (11), we get

Rex1/2Cf=f″(0),Rex−1/2Nux=−θ′(0) (15)

where Re_*x*_ = u_w_(x)x/*v* is the local Reynolds number.

### Flow stability

It has been shown in some papers (Weidman et al. [52], Roşca and Pop [50], Ishak [53] etc.) that dual (lower and upper branch) solutions exist. In order to determine which of these solutions are stable and physically realizable, a stability analysis to the solutions of Eqs. (1)–(5) needs to done. Thus, the new variables for the unsteady problem are

$$\begin{array}{l} \psi =\sqrt{c\nu }xf(\eta ,\tau ),\;u=cx\frac{\partial f}{\partial \eta }(\eta ,\tau ),\;v=-\sqrt{c\nu }f(\eta ,\tau ),\\ \theta (\eta ,\tau )=(T-T_{\infty })/(T_{w}-T_{\infty }),\;\phi (\eta ,\;\tau )=(C-C_{\infty })/(C_{w}-C_{\infty }),\\ \tau =at,\;\eta =\sqrt{c/\nu }y. \end{array}$$ (16)

Therefore, Eqs. (2)–(4) can be written as

$$\frac{\partial^{3}f}{\partial \eta^{3}}+f\frac{\partial^{2}f}{\partial \eta^{2}}-{\left(\frac{\partial f}{\partial \eta }\right)}^{2}+M\left(A-\frac{\partial f}{\partial \eta }\right)+A^{2}-\frac{\partial^{2}f}{\partial \eta \partial \tau }=0,$$ (17)

$$\frac{1}{\mathrm{Pr}}\frac{\partial^{2}\theta }{\partial \eta^{2}}+f\frac{\partial \theta }{\partial \eta }+Nb\frac{\partial \phi }{\partial \eta }\frac{\partial \theta }{\partial \eta }+Nt{\left(\frac{\partial \theta }{\partial \eta }\right)}^{2}-\frac{\partial \theta }{\partial \tau }=0,$$ (18)

$$\frac{\partial^{2}\phi }{\partial \eta^{2}}+Lef\frac{\partial \phi }{\partial \eta }+\frac{Nt}{Nb}\frac{\partial^{2}\theta }{\partial \eta^{2}}-\frac{\partial \phi }{\partial \tau }=0$$ (19)

and are subjected to the boundary conditions

$$\begin{array}{l} f(0,\tau )=S,\;\frac{\partial f}{\partial \eta }(0,\tau )=\lambda ,\;\theta (0,\tau )=1,\;\phi \left(0,\tau \right)=1,\\ \frac{\partial f}{\partial \eta }(\eta ,\tau )\to A,\;\theta (\eta ,\tau )\to 0,\;\phi (\eta ,\tau )\to 0\;\text{as}\;\eta \to \infty . \end{array}$$ (20)

The following expressions are written to check the stability of the steady flow solution *f*(*η*) = *f*_0_(*η*), *θ*(*η*) = *θ*_0_(*η*) and *ϕ*(*η*) = *ϕ*_0_(*η*) that satisfy the boundary-value problem (8)–(10) (see [50], [52], [53]),

$$\begin{array}{c} f(\eta ,\tau )=f_{0}(\eta )+e^{-\gamma \tau }\;F(\eta ,\tau ),\;\theta (\eta ,\tau )=\theta_{0}(\eta )+e^{-\gamma \tau }\;G(\eta ,\tau ),\\ \phi \left(\eta ,\tau \right)=\phi_{0}\left(\eta \right)+e^{-\gamma \tau }H\left(\eta ,\tau \right) \end{array}$$ (21)

where *γ* is an unknown eigenvalue parameter, and *F*(*η*,*τ*), *G*(*η*,*τ*) and *H*(*η*,*τ*) are small as compared to *f*_0_(*η*), *θ*_0_(*η*) and *ϕ*_0_(*η*). By substituting (21) into Eqs. (17)–(19), the following linearized problems are obtained:

$$\frac{\partial^{3}F}{\partial \eta^{3}}+f_{0}\frac{\partial^{2}F}{\partial \eta^{2}}+f_{0}{}^{\prime \text{​}\prime }F-\left(2f_{0}{}^{\prime }-\gamma \right)\frac{\partial F}{\partial \eta }+M\left(A-\frac{\partial F}{\partial \eta }\right)-\frac{\partial^{2}F}{\partial \eta \partial \tau }=0,$$ (22)

$$\frac{1}{\mathrm{Pr}}\frac{\partial^{2}G}{\partial \eta^{2}}\;+\left(f_{0}+Nb\phi_{0}^{\prime }+2Nt\theta_{0}^{\prime }\right)\frac{\partial G}{\partial \eta }+\theta_{0}^{\prime }F+\gamma G+Nb\theta_{0}^{\prime }\frac{\partial H}{\partial \eta }-\frac{\partial G}{\partial \tau }=0,$$ (23)

$$\frac{\partial^{2}H}{\partial \eta^{2}}+Le\left(f_{0}\frac{\partial H}{\partial \eta }+\phi_{0}^{\prime }F\right)+\frac{Nt}{Nb}\frac{\partial^{2}G}{\partial \eta^{2}}+\gamma H-\frac{\partial H}{\partial \tau }=0.$$ (24)

The boundary conditions are

$$\begin{array}{l} F(0,\tau )=0,\;\frac{\partial F}{\partial \eta }(0,\tau )=0,\;G\left(0,\tau \right)=0,\;H\left(0,\tau \right)=0,\\ \frac{\partial F}{\partial \eta }(\eta ,\tau )\to 0,\;G(\eta ,\tau )\to 0,\;H\left(\eta ,\tau \right)\to 0\;\text{as}\;\eta \to \infty . \end{array}$$ (25)

We set *τ* = 0 to get the solution *f*(*η*) = *f*_0_(*η*), *θ*(*η*) = *θ*_0_(*η*) and *ϕ*(*η*) = *ϕ*_0_(*η*) of the steady equations (8)–(10). Hence *F* = *F*_0_(*η*), *G* = *G*_0_(*η*) and *H* = *H*_0_(*η*) in (22)–(24) identify initial growth or decay of the solution (21). Therefore, the following linear eigenvalue problems have to solved:

$$F_{0}^{\prime \prime \prime }+f_{0}F_{0}^{\prime \prime }+f_{0}^{\prime \prime }F_{0}-\left(2f_{0}^{\prime }-\gamma \right)F_{0}^{\prime }+M\left(A-F_{0}^{\prime }\right)=0,$$ (26)

$$\frac{1}{\mathrm{Pr}}G_{0}^{\prime \prime }+\left(f_{0}+Nb\phi_{0}^{\prime }+2Nt\theta_{0}^{\prime }\right)G_{0}^{\prime }+\theta_{0}^{\prime }F_{0}+Nb\theta_{0}^{\prime }H_{0}^{\prime }+\gamma G_{0}=0,$$ (27)

$$H_{0}^{\prime \prime }+Le\left(f_{0}H_{0}^{\prime }+\phi_{0}^{\prime }F_{0}\right)+\frac{Nt}{Nb}G_{0}^{\prime \prime }+\gamma H_{0}=0$$ (28)

along with the boundary conditions

$$\begin{array}{l} F_{0}(0)=0,F_{0}^{\prime }(0)=0,G_{0}(0)=0,H_{0}\left(0\right)=0,\\ F_{0}^{\prime }(\eta )\to 0,G_{0}(\eta )\to 0,H_{0}(\eta )\to 0\;\text{as}\;\eta \to \infty . \end{array}$$ (29)

The smallest eigenvalue *γ* will determine the stability of the corresponding steady flow solution *f*_0_(*η*), *θ*_0_(*η*) and *ϕ*_0_(*η*) for all parameters involved. Harris et al. [54] suggests relaxing a boundary condition on *F*_0_(*η*) or *G*_0_(*η*) to better find the range of possible eigenvalues. Hence, for the present problem, we relax the condition that $F_{0}^{\prime }(\eta )\to 0$ as *η* → ∞ and for a fixed value of *γ* we solve the system (26, 27, 28) along with the new boundary condition$F_{0}^{\prime \prime }(0)=1$.

## Results and Discussions

The system of ordinary differential equations (8)–(10) subject to the boundary conditions (11) was solved numerically for different values of parameters: the stretching/shrinking parameter *λ*, suction parameter *S*, magnetic parameter *M*, velocity ratio parameter *A*, Brownian motion parameter *Nb* and thermophoresis parameter *Nt*. The Prandtl number *Pr* is set equal to 6.8 (water at 20°C) throughout the paper. The relative tolerance is set to 10^–10^. The boundary conditions (11) at *η* = ∞ are replaced by *η* = 10. This choice is adequate for the velocity and temperature profiles to reach the far field boundary conditions asymptotically. The function bvp4c in MATLAB is used to solve the equations due to its effectiveness in solving the boundary value problems which are much harder than initial value problems. However, the ordinary differential equations (8)–(10) need to be reduced to first order ordinary differential equations before solving them. These procedures are explained in detail by Shampine et al. [55, 56]. We note that for *S* = *λ* = *M* = 0 and *A* = 1, the skin friction coefficient (*f*″(0) = 1.232587) obtained is in excellent agreement with the result reported by Bejan [57] which gives rise to the confidence in the validity of our results.

**Fig 2:**
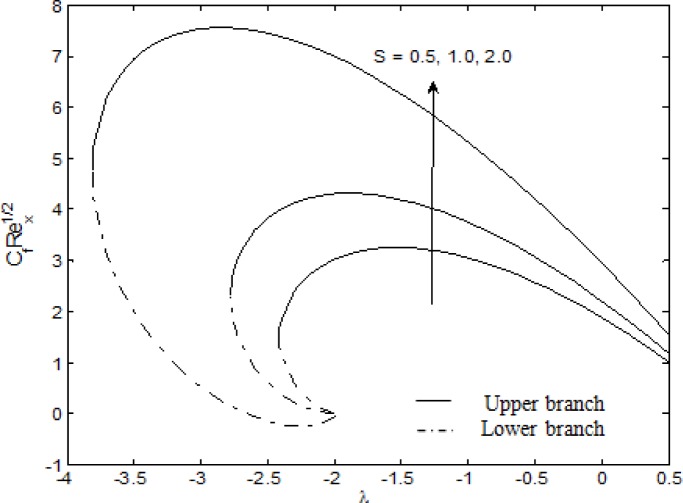
Variation of the skin friction coefficient with *λ* for different values of *S* when *M* = *A* = 1.

In this paper, we investigated the MHD stagnation point flow of a nanofluid over a stretching/shrinking sheet in the presence of suction effect at the boundary. Fig. 2 shows the skin friction coefficient for different suction parameter *S* where we can see that the range of solutions depend on *S* and the stretching/shrinking parameter *λ*. From Fig. 2, it is seen that for all values of *S*, unique solution exists for *λ* > ˗2 and *λ* = *λ*_*c*_ where *λ*_*c*_ is the critical values of *λ* when *M* = *A* = 1. Furthermore, it is noted that the range of *λ*, where solutions exist, increases as *S* increases as seen in Table 1. As suction increases, the vorticity will continue to be confined even for larger *λ*. As a result, the range of existence of solution increases [42, 58]. In addition, the range of solutions is also affected by the change in magnetic parameter *M* and velocity ratio parameter *A*. For future reference, we also include the values of *λ*_*c*_ for different values of *M* and *A* in Table 1.

**Table 1:** Values of *λ*_*c*_.

*S*	*M*	*A*	*λ*_*c*_
0.5	1.0	1.0	−2.4142
1.0			−2.7755
2.0			−3.8116
0.5	1.1		−2.5060
	1.2		−2.5508
	1.0	1.1	−2.5100
		1.2	−2.6003

The effect of suction, magnetic, velocity ratio and stretching/shrinking parameters on the skin friction coefficient are displayed in Fig. 2 and Table 2. The skin friction coefficient decreases as *λ* increases. However, it increases with increasing *S*, *M* and *A*. From Fig. 2, it is observed that the differences between the skin friction coefficient grow as the sheet keeps shrinking. From the lower branch, the skin friction coefficient is seen to approach 0 as *λ* approaches-2.

**Table 2:** Variation of the skin friction coefficient for different values of *M* and *A* when *S* = 0.5 and *λ* = −1.

*M*	*A*	*f″*(0)
1.0	1.0	1.8285
1.1		2.4545
1.2		2.8287
1.0	1.1	2.7393
	1.2	3.3264

**Fig 3:**
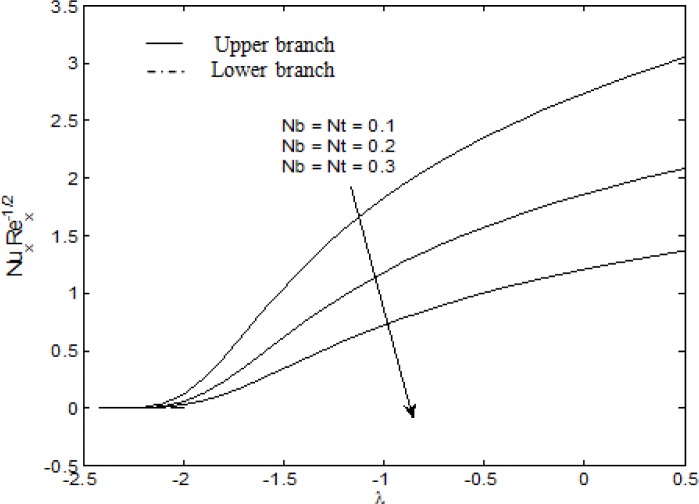
Variation of the local Nusselt number with *λ* for different values of *Nb* and *Nt* when *S* = 0.5, *M* = *A* = 1, *Pr* = 6.8 and *Le* = 2.

Fig. 3 shows the effects of Brownian motion and thermophoresis parameter on the surface heat transfer rate (local Nusselt number). The local Nusselt number increases with *λ*. However, as *Nb* and *Nt* increase, the local Nusselt number decreases. This is due to the fact that the thermal boundary layer thickness will increase once *Nb* and *Nt* are intensified. As the thermal boundary layer thickness grows larger, we can expect that the temperature gradient at the surface grows smaller [51]. Thus, the local Nusselt number decreases. It is interesting to note that the local Nusselt number for the three different values of *Nb* and *Nt* are shown to converge to approximately 0 at *λ* = *λ*_*c*_. Furthermore, similar to Fig. 2, from the lower branch, it is seen that the local Nusselt number approaches 0 as *λ* approaches-2.

**Fig 4:**
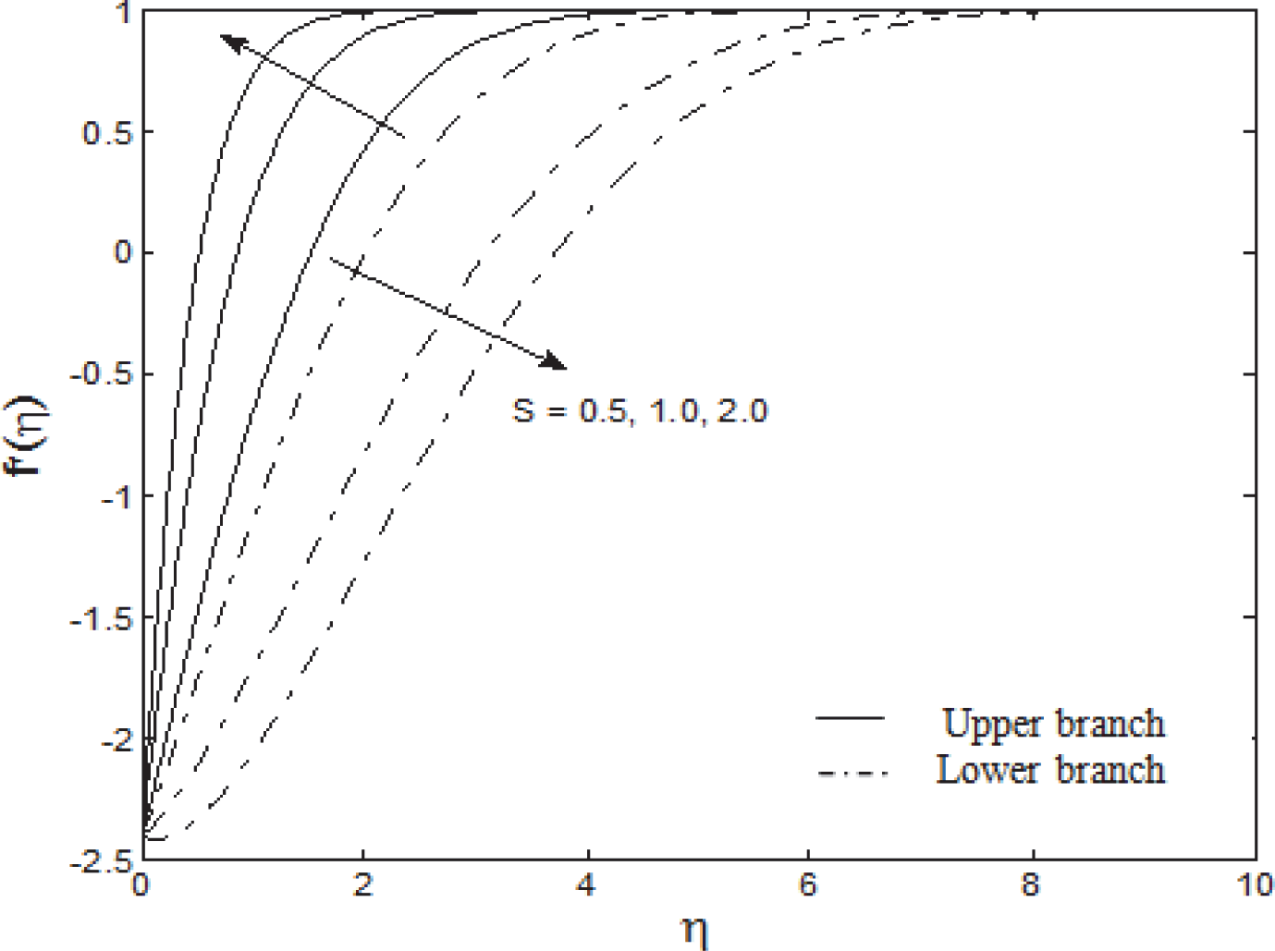
The velocity profiles for different values of *S* when *M* = *A* = 1 and *λ* = −2.4.

**Fig 5:**
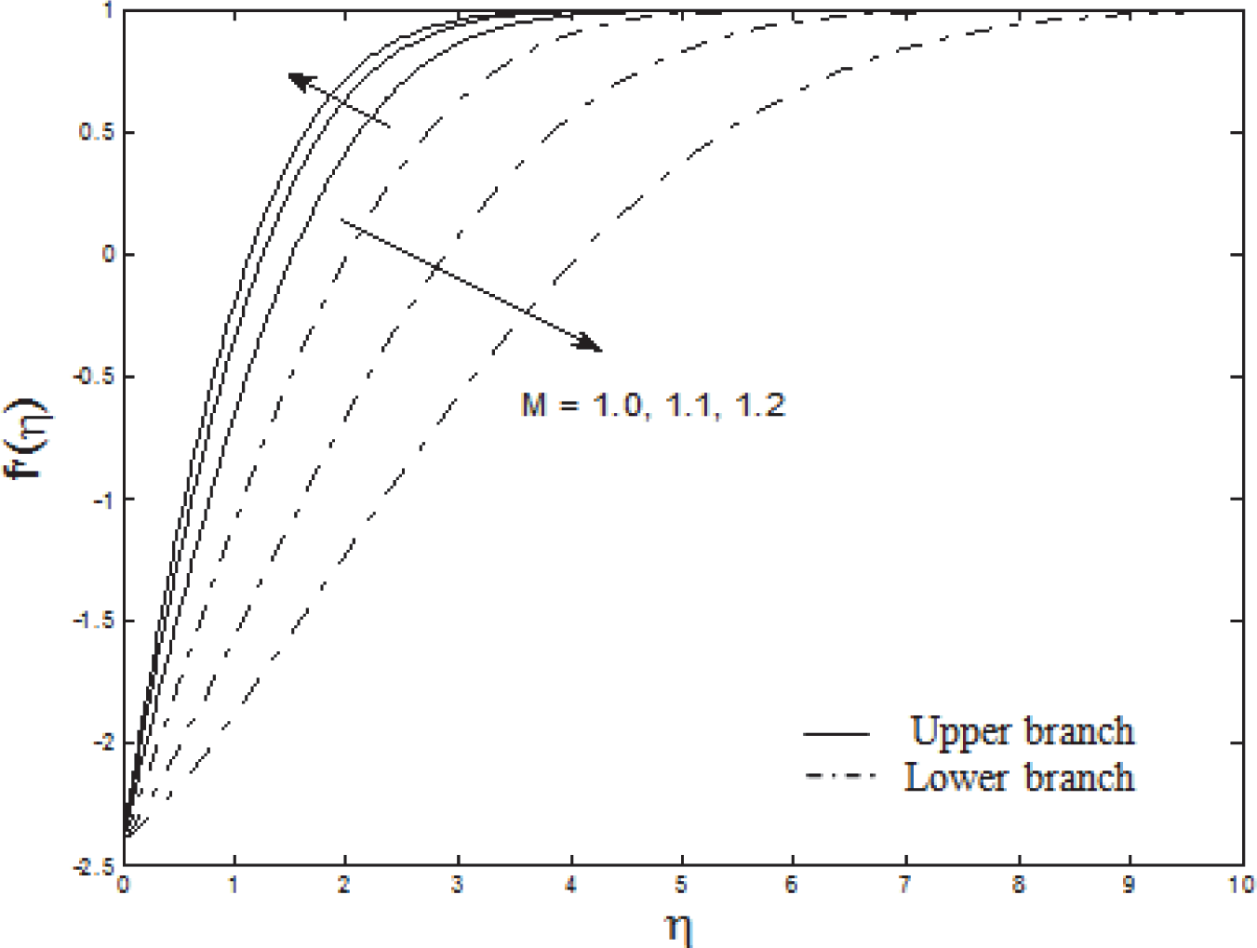
The velocity profiles for different values of *M* when *S* = 0.5, *A* = 1 and *λ* = −2.4.

**Fig 6:**
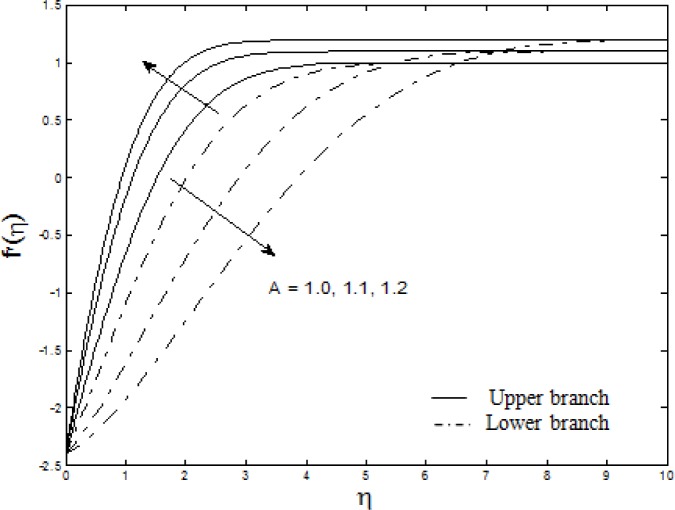
The velocity profiles for different values of *A* when *S* = 0.5, *M* = 1 and *λ* = −2.4.

**Fig 7:**
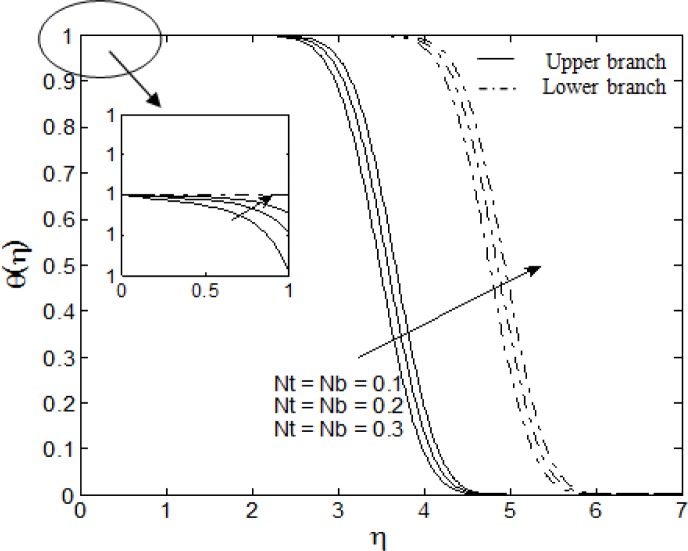
The temperature profiles for different values of *Nb* and *Nt* when *S* = 0.5, *M* = *A* = 1, Pr = 6.8, Le = 2 and *λ* = −2.4.

Figs. 4–7 depict the samples of velocity and temperature profiles for different values of *S*, *M*, *A* and *Nt = Nb*. These profiles satisfy the far field boundary conditions (11) asymptotically, which support the validity of the results obtained, as well as supporting the existence of dual solutions shown in Figs. 2 and 3. The velocity profiles in Fig. 4 show that as suction increases, the velocity increases for upper branch and decreases for lower branch. Furthermore, it is observed that with the increasing suction, the boundary layer thickness decreases for the upper branch while it increases for the lower branch. This phenomenon concurs with Fig. 2 (at *λ* = −2.4) where the skin friction coefficient increases for upper branch and decreases for lower branch as suction increases. Figs. 5 and 6 show the effects of magnetic parameter *M* and velocity ratio parameter *A* on the velocity for the shrinking sheet. It is seen that for the increasing *M* and *A*, the velocity also increases. The profiles generated are qualitatively similar to those of Ibrahim et al. [51]. Also, the boundary layer thicknesses decrease as both *M* and *A* increase. In Fig. 7, the temperature profiles generated show that the temperature increases as *Nt* and *Nb* increase. Furthermore, the profiles show that as the boundary layer thickness increases with the increasing *Nt* and *Nb*, the temperature gradient decreases and thus reducing the local Nusselt number shown in Fig. 3.

The numerical results show that dual solutions exist for Eqs. (8)–(10) with boundary conditions (11) when *λ≤* −2. It is our intention to show that only the upper branch solutions are stable and physically realizable, while the lower branch solutions are not stable, and hence, not physically realizable. According to Weidman [52] and Merkin [59], as the eigenvalue *γ* = 0, Eqs. (26)–(28) yield the homogeneous problem which defines the critical value *λ*_*c*_, which is the turning point value that separates the stable and unstable branches. The sign of *γ* will change at the turning point. Therefore, we solved the eigenvalue problem (26)–(28) to determine the smallest eigenvalues *γ* on the upper and lower solution branches. These results are displayed in Table 3 for several values of *λ* when *S* = 0.5 and *M* = *A* = 1. The results show that positive values of *γ* are obtained at the upper branch while negative values of *γ* are found at the lower branch, showing that the values of *γ* change from positive (stable) to negative (unstable) at the turning points of the curves, which, as shown in Table 1, is *λ*_*c*_ = −2.4142. It is also noted that for both upper and lower branches, |*γ*| decreases as *λ* approaches *λ*_*c*_ which confirms the observation of Merkin [59] where *γ* = 0 at *λ* = *λ*_*c*_.

**Table 3:** The smallest eigenvalue *γ* when *S* = 0.5 and *M* = *A* = 1.

*Λ*	*γ*	*γ*
	(Upper branch)	(lower branch)
−2.400	3.7393	−2.7709
−2.410	3.5737	−2.6594
−2.414	3.4331	−2.5509

## Conclusions

The magnetohydrodynamic (MHD) stagnation point flow of a nanofluid over a stretching/shrinking sheet with suction is studied. Numerical results were obtained using the function bvp4c in MATLAB for several range of parameters: suction parameter, magnetic parameter, velocity ratio parameter, stretching/shrinking parameter, and Brownian motion and thermophoresis parameters. The results show that dual solutions exist. The range of the solution domain increases with increasing the suction effect at the boundary as well as the presence of magnetic field. Furthermore, increasing suction as well as the magnetic effect causes the skin friction coefficient to increase. Increasing the Brownian motion and thermophoresis parameters lower the local Nusselt number.
